# Serum Catestatin Concentrations Are Increased in Patients with Atrial Fibrillation

**DOI:** 10.3390/jcdd10020085

**Published:** 2023-02-17

**Authors:** Josip Katic, Zrinka Jurisic, Marko Kumric, Josip A. Borovac, Ante Anic, Toni Breskovic, Daniela Supe-Domic, Josko Bozic

**Affiliations:** 1 Department of Cardiology, University Hospital of Split, 21000 Split, Croatia; 2 Department of Internal Medicine, University of Split School of Medicine, 21000 Split, Croatia; 3 Department of Pathophysiology, University of Split School of Medicine, 21000 Split, Croatia; 4 Department of Health Studies, University of Split, 21000 Split, Croatia; 5 Department of Medical Laboratory Diagnostics, University Hospital of Split, 21000 Split, Croatia

**Keywords:** catestatin, atrial fibrillation, autonomic nervous system, atrial remodeling

## Abstract

The autonomic nervous system is crucial in initiating and maintaining atrial fibrillation (AF). Catestatin is a multipurpose peptide that regulates cardiovascular systems and reduces harmful, excessive activity of the sympathetic nervous system by blocking the release of catecholamines. We aimed to determine whether serum catestatin concentrations are associated with AF severity, duration indices, and various clinical and laboratory indicators in these individuals to better define the clinical value of catestatin in patients with AF. The present single center study enrolled 73 participants with AF and 72 healthy age-matched controls. Serum catestatin concentrations were markedly higher in AF patients than controls (14.11 (10.21–26.02) ng/mL vs. 10.93 (5.70–20.01) ng/mL, *p* = 0.013). Furthermore, patients with a more severe form of AF had significantly higher serum catestatin (17.56 (12.80–40.35) vs. 10.98 (8.38–20.91) ng/mL, *p =* 0.001). Patients with higher CHA_2_DS_2_-VASc scores (17.58 (11.89–37.87) vs. 13.02 (8.47–22.75) ng/mL, *p* = 0.034) and higher NT-proBNP levels (17.58 (IQR 13.91–34.62) vs. 13.23 (IQR 9.04–22.61), *p* = 0.036) had significantly higher serum catestatin concentrations. Finally, AF duration correlated negatively with serum catestatin levels (r = −0.348, *p* = 0.003). The results of the present study implicate the promising role of catestatin in the intricate pathophysiology of AF, which should be explored in future research.

## 1. Introduction

Atrial fibrillation (AF) is a major clinical problem involving many patients with different arrhythmia-related symptoms and causing a significant social and economic impact. From a clinical point of view, AF has been classified as paroxysmal or persistent about the modality of termination and permanent when the efforts to restore sinus rhythm become ineffective [1]. It is a common opinion that the time frame elapsing from the paroxysmal to the permanent form may be highly variable and influenced by several factors, such as the presence or absence of structural heart disease, atrial dimension, comorbidities, and the duration of the arrhythmia [2]. All these factors are recognized as significant determinants of the extent of electrical, mechanical, and anatomical remodeling [2]. The incidence of atrial fibrillation could be predicted most accurately using a combination of biomarkers, common genetic variation, and well-established classical cardiovascular risk factors (CVRF) [3].

In the last decade, the autonomic nervous system (ANS) has emerged as an essential mechanism in initiating and maintaining AF, with both sympathetic and parasympathetic innervation being pro-arrhythmic in the atria [4]. In humans and animal models with AF, there was an increase in sympathetic nerve density [4,5]. A histological study of the human pulmonary vein (PV)-left atrium (LA) junction showed numerous autonomic nerves [6]. Greater nerve densities are found in the epicardium than the endocardium, and they are located in the left atrium, specifically within 5 mm of the PV-LA junction [6]. In addition to the complex anatomic and physiological interactions between various nerve structures, cardiac autonomic innervation is constantly remodeling, especially during disease states. Increased atrial sympathetic innervation is associated with increased incidence and duration of AF in animal models [7,8]. Consistent with these results, atrial sympathetic nerve densities significantly increase in patients with permanent AF [9]. The effect of heightened sympathetic activity in the atria is complex but mainly occurs through activating the beta-adrenergic receptor, a G-protein coupled receptor. Intracellular signaling involves the activation of protein kinase A (PKA), which leads to a wide range of effects through the phosphorylation of target proteins. The predominant effect of adrenergic activation is enhanced Ca^2+^ handling, which promotes focal activity, though early or delayed afterdepolarizations [10].

In 1997, Mahata et al. identified catestatin, a 21-amino-acid product of chromogranin A (ChgA) [11]. ChgA is released by exocytosis upon sympathetic axons or chromaffin cell stimulation. Pro-protein convertases process ChgA extracellularly upon translation, releasing a variety of bioactive polypeptides, including catestatin [12]. Mahapatra and colleagues demonstrated that catestatin inhibited nicotinically triggered exocytotic release of several co-transmitters from chromaffin granules such as neuropeptide Y, adenosine triphosphate, chromogranins, and catecholamines, thereby indicating that catestatin is a potent regulator of neuropeptide transmission in the sympathochromaffin system [13]. Of established cardiovascular effects, catestatin suppresses beta-adrenergic activation and acts in a negative inotropic and chronotropic fashion, stimulates angiogenesis and proliferation of vascular smooth muscle cells, decreases endothelial cell thrombogenicity, suppresses endothelial cells, and suppresses atherosclerosis and inflammation, while also exerting cardioprotective effects by abrogating cardiomyocyte ischemia-reperfusion injury [14].

The potential role of catestatin, as a potent neuropeptide transmission regulator in the sympathochromaffin system, in the pathophysiology of AF is largely unexplored. Therefore, in the present study, we aimed to examine if serum catestatin levels will differ between patients with AF and matched healthy controls. Furthermore, we sought to establish whether serum catestatin levels will be associated with AF severity and duration and with relevant clinical and laboratory parameters in this population.

## 2. Materials and Methods

### 2.1. Study Design and Ethical Considerations

The present study was designed as cross-sectional and included consecutive and scheduled paroxysmal AF (PAF), persistent AF (PersAF), and long-persistent for electric direct-current cardioversion (DC), and ablation treatment of AF at the Cardiovascular Diseases Department, University Hospital Split, Croatia, from October 2021 to August 2022. Antiarrhythmic medications that were used in the present study include beta blockers, amiodarone, propafenone, calcium channel blockers, and digoxin. The control group was constituted of age- and sex-matched controls that had no history of AF or other significant supraventricular or ventricular arrhythmias or antiarrhythmic medication prescribed. All subjects with permanent atrial fibrillation, advanced structural heart disease (including moderate to severe valvular disease, hypertrophic or dilated cardiomyopathy, and congenital heart disease), uncontrolled thyroid or parathyroid disease, advanced chronic liver, kidney, or lung disease, systemic or local inflammatory or infectious disease, contraindication for anticoagulation, pregnancy, and malignancy were excluded from the study. Informed written consent was obtained from each participant enrolled in the study. In contrast, the study protocol was approved by the Ethics Committee of the University Hospital of Split (Class: 500-03/21-01/99, Number: 2181-147/01/06/M.S.-21-02). The study adhered to the ethical guidelines in the Declaration of Helsinki as reflected in a priori approval by the institution’s Ethics Committee.

### 2.2. Clinical and Laboratory Evaluations

For each patient, detailed data on medical history were obtained. Patients underwent a physical examination, 12-lead electrocardiogram (ECG) recording, and baseline anthropometric measurements. For the body weight (kg) and height (cm) measurements, the calibrated scale was used (Seca, Birmingham, UK). At the same time, body mass index was calculated by the following formula: body weight (kg) divided by height squared (m^2^).

The AF types were defined by the contemporary current European Society of Cardiology (ESC) guidelines [1]. The physician categorized AF-related symptoms according to the European Heart and Rhythm Association (EHRA) classification as class I (no evident symptoms signs), class II (mild symptoms: regular daily activity not affected), class III (severe symptoms: regular daily activity affected), or as class IV (disabling symptoms: regular daily exercise discontinued) [1]. According to the ESC guidelines, thromboembolic and hemorrhagic risk stratifications were calculated by using CHA2DS2VASc and HAS-BLED scores [1]. In addition, all included patients have been treated according to contemporary evidence-based medicine and guidelines [1].

We collected patients’ clinical data, including patient history, transthoracic echocardiography measurements, and 24-h Holter ECG. Transthoracic echocardiography was performed using Vivid IQ (GE HealthCare, Chicago, IL, USA) in every patient. An experienced echocardiographer obtained all echocardiographic measurements. At the same time, patients were in left lateral decubitus, following recommendations for cardiac chamber quantification issued by the European Association of Cardiovascular Imaging (EACVI) [15]. Left ventricular ejection fraction (LVEF) was measured by Simpson’s 2D biplane method. The left atrial diameter was measured in the parasternal long-axis view. The 24-h Holter ECG was analyzed by an experienced electrophysiologist subject’s group allocation.

In all participants, fasting laboratory tests were drawn at admission. Venous blood samples were obtained from the antecubital vein in each subject after fasting for 12 h. All blood samples were analyzed following good standards of laboratory practice in the same biochemical laboratory and by the same specialist in medical biochemistry that was blinded to the subject’s assignment in the study groups. 

Catestatin (Cat. no. EK-053-27CE, EIA kit, Phoenix Pharmaceuticals Inc., Burlingame, CA, USA) concentrations in serum were determined by an enzyme-linked immunosorbent assay (ELISA) with reported sensitivity for catestatin of 0.05 ng/mL with a linear range of 0.05–0.92 ng/mL. The reported cross-reactivity with endogenous human catestatin peptide for the used assay kit was 100%, with an intra-assay coefficient of variability (CV) < 10% and an inter-assay CV < 15%.

### 2.3. Statistical Analysis

MedCalc Statistical Software version 20.113 (MedCalc Software BV, Ostend, Belgium) and GraphPad Prism version 9.4.1 (GraphPad Software, Inc., San Diego, CA, USA) were used for statistical data analysis and graph design. Qualitative data were expressed as numbers and percentages, and quantitative data as mean ± standard deviation (SD) or median and interquartile range (IQR), based on normality distribution. The Kolmogorov–Smirnov test was used to assess the normality of the data distribution. Qualitative variables were analyzed using the Chi-squared (χ^2^) test. Quantitative variables were compared using Student’s *t*-test for independent samples or Mann–Whitney U test, as appropriate. Patients with AF were subdivided and analyzed using several distinct criteria: CHA2DS2-VASc score, EHRA score, N-terminal pro brain natriuretic peptide (NT-proBNP) concentration, and the presence of beta-blocker therapy. The Kruskal–Wallis test was employed in analyses in which catestatin was compared between 3 or more variables. Finally, for correlation analysis between catestatin and other parameters, the Spearman rank correlation was used. Statistical significance was set at *p* < 0.05 for all comparisons.

## 3. Results

The present study included 73 adult patients with pre-diagnosed AF (26 patients with paroxysmal, 34 patients with persistent, and 13 patients with long-persistent AF), and 72 healthy, age- and sex-matched controls. The baseline characteristics of patients are shown in Table 1. Laboratory analyses showed that the AF group compared with the control group had significantly lower total cholesterol (5.0 (4.1–6.1) vs. 5.6 (4.6–6.3), *p* = 0.027) and LDL (2.8 (2.1–4.0) vs. 3.5 (2.7–4.1) mmol/L, *p* = 0.014), while they had significantly higher triglycerides levels (1.4 (1.1–1.9) vs. 1.1 (0–7–1.9) mmol/L, *p* = 0.026).

**Figure 1:**
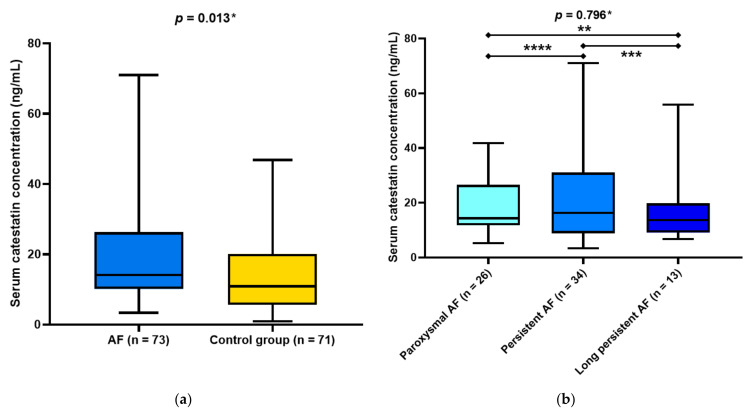
(**a**) Comparison of catestatin concentrations between patients with atrial fibrillation and healthy controls. Data are presented as median and interquartile range. * Mann–Whitney U test. (**b**) Comparison of catestatin concentrations between subgroups of patients with atrial fibrillation. Data are presented as median and interquartile range. * Kruskal–Wallis test ** *p* = 0.447 *** *p* = 0.634 **** *p* = 0.911.

Serum catestatin levels were significantly higher in the AF group compared with the control group (14.11 (10.21–26.02) vs. 10.93 (5.70–20.01) ng/mL, *p* = 0.013) (Figure 1a). Furthermore, there were no statistically significant differences in serum catestatin levels between the patients with clinical types of AF (14.30 (11.94–25.70) vs. 16.26 (8.90–29.12) vs. 13.65 (9.10–19.18), *p* = 0.796) (Figure 1b).

**Figure 2:**
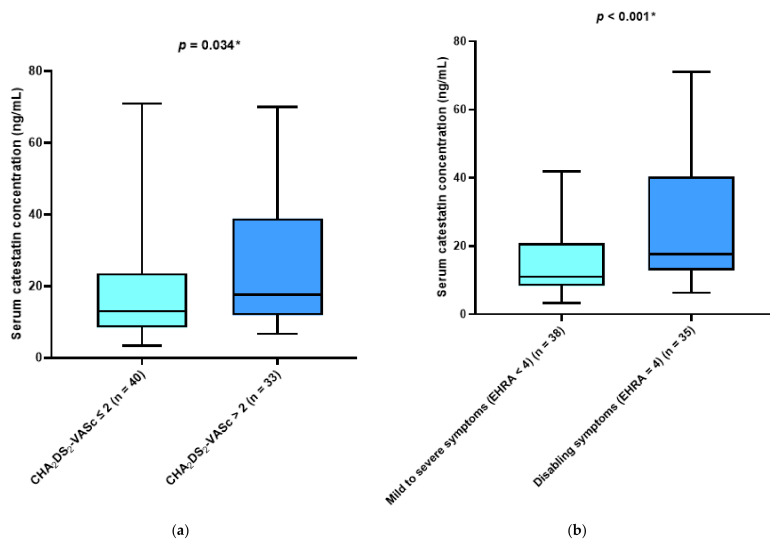
(**a**) Comparison of serum catestatin concentrations with respect to CHA2DS2-VASc score. Data are presented as median and interquartile range. * Mann–Whitney U test. (**b**) Comparison of serum catestatin concentrations with respect to the severity of symptoms. Data are presented as median and interquartile range. * Mann–Whitney U test.

There was a significant difference in median catestatin levels between patients with CHA2DS2-VASc scores > 2 compared with patients with CHA2DS2-VASc scores ≤ 2 (17.58 (11.89–37.87) vs. 13.02 (8.47–22.75) ng/mL, *p* = 0.034) (Figure 2a). Furthermore, the participants’ group with EHRA score four criteria had significantly higher median catestatin levels compared with patients with EHRA score ≤ 3 (17.56 (12.80–40.35) vs. 10.98 (8.38–20.91) ng/mL, *p* < 0.001) (Figure 2b).

**Figure 3:**
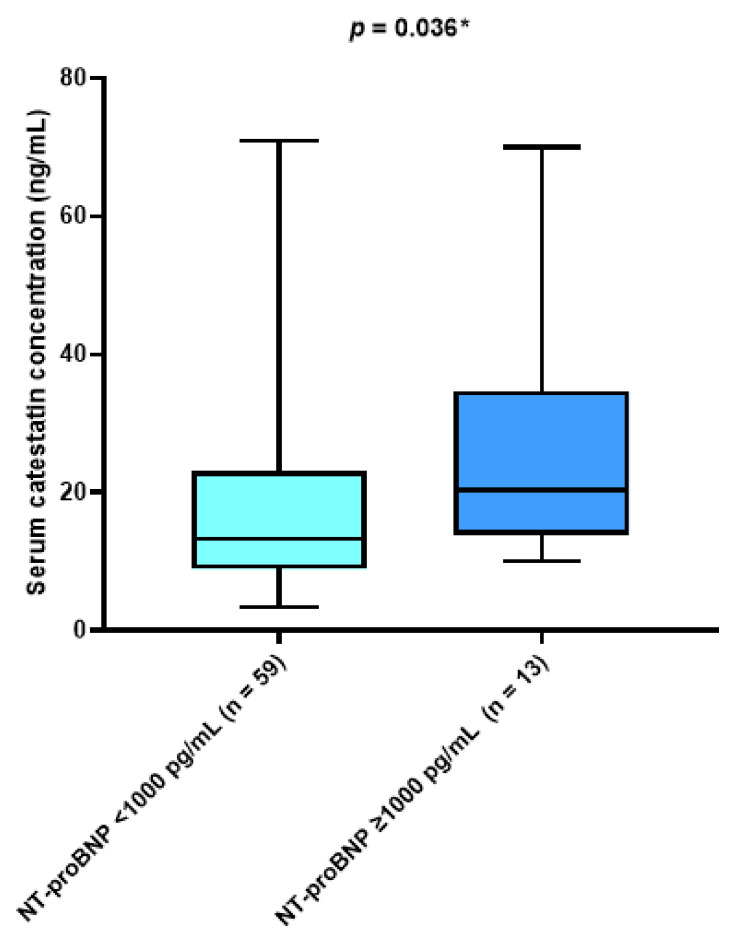
Comparison of serum catestatin concentrations with respect to NT-proBNP levels. Data are presented as median and interquartile range. * Mann–Whitney U test.

There was a statistically significant difference in median catestatin level between patients with an NT-proBNP above 1000 pg/mL compared with patients with an NT-proBNP concentration below 1000 pg/mL (17.58 (13.91–34.62) vs. 13.23 (9.04–22.61) ng/mL, *p* = 0.036) (Figure 3).

**Figure 4:**
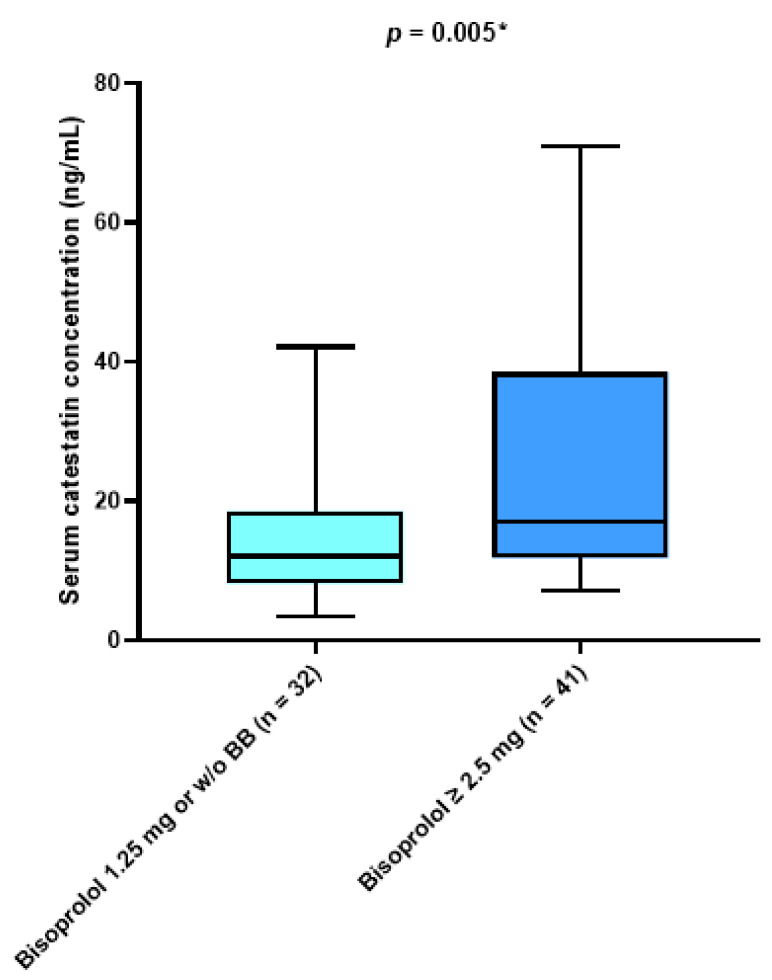
Comparison of serum catestatin concentrations with respect to beta-blocker therapy. Data are presented as median and interquartile range. * Mann–Whitney U test.

**Figure 5:**
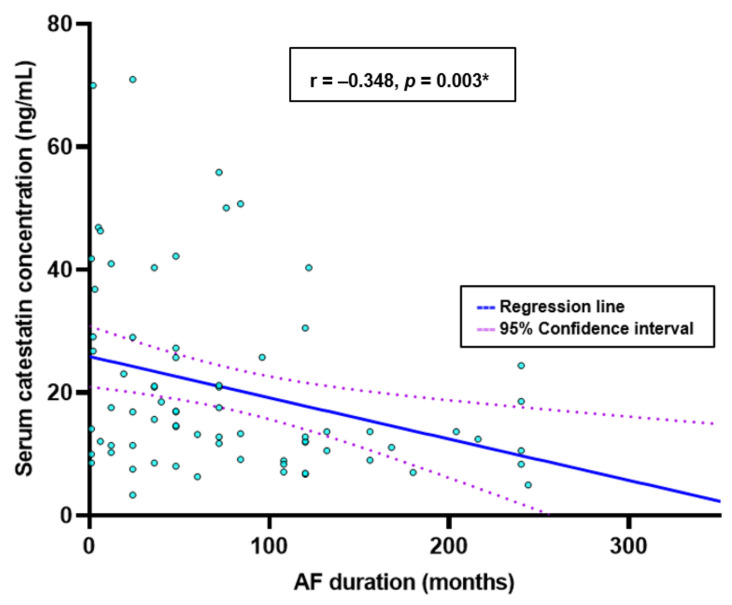
Correlation between serum catestatin concentrations and duration of the disease. Data are presented as median and interquartile range. * Spearman rank correlation analysis.

Patients treated with a higher dose of beta blockers (≥ 2.5 mg daily) and those treated with a beta-blocker dose less than 2.5 mg daily or not treated at all were significantly different in median catestatin value (17.01 (11.89–37.71) vs. 12.09 (8.21–18.55), *p* = 0.005) (Figure 4). There was a significant negative correlation between serum catestatin levels and the duration of the AF in months (r = −0.348, *p* = 0.003) (Figure 5).

## 4. Discussion

To the best of our knowledge, this is the first study investigating the catestatin level in AF patients. We found that serum catestatin levels were significantly higher in AF patients than in normal sinus rhythm (NSR) controls. Furthermore, catestatin concentrations were higher among those included with higher severity of AF (as assessed by EHRA score) and thromboembolic risk (as assessed by CHA2DS2-VASc score). Our findings also revealed a negative correlation between serum catestatin concentrations and AF duration. Similarly, we show that higher doses of beta blockers were associated with greater circulating catestatin levels. Similarly, patients with AF and greater baseline neurohumoral activity, as evidenced by increased NT-proBNP levels, had overall greater circulating catestatin levels, and this was particularly evident in the group of AF patients with NT-proBNP > 1000 pg/mL. 

Assessing sympathetic nervous system (SNS) activity in patients with atrial fibrillation presents a unique challenge. Currently, no method for SNS activity assessment can be regarded as the “gold standard.” Laboratory biomarkers that can be measured in AF patients’ peripheral circulation seem to be implicated in the pathophysiology of SNS activation in AF. Biomarkers were found to be differentially expressed between women and men with AF, suggesting that the pathophysiological mechanisms underlying AF may vary and all-cause mortality and major adverse cardiovascular events could be predicted by certain biomarkers [16,17]. For the first time, the present work provides information on circulating catestatin levels in the AF population. Specifically, in the present study, we found catestatin to be significantly higher than in healthy subjects, which could indicate the presence of augmented cardiac sympathetic activation. In the last decade, the ANS has emerged as a critical mechanism in initiating and maintaining AF. The β-adrenergic receptor (β-AR)/cyclic adenosine monophosphate (cAMP) pathway is a major pathway in the sympathetic regulation of heart function [18]. Catestatin’s physiological action inhibits catecholamine release into circulation via a noncompetitive and reversible antagonism of neuronal nicotinic cholinergic receptors (nAChR) [13]. Catestatin blunted the stimulatory effects of norepinephrine and other mitogenic signals on 1- and 2-adrenergic receptors, providing novel evidence that catestatin modulates adrenergic transmission at the receptor level [19]. Catestatin reduces both inotropy and lusitropy under basal and stimulated (1adrenergic and endothelin) circumstances in a Langendorff-perfused rat heart preparation, providing evidence that catestatin operates as a cardioprotective peptide [20]. Intramyocardial ChgA production in humans is related to deleterious inotropic effects on the mammalian heart, indicating neuroendocrine regulation of cardiac function by ChgA [21]. Several findings suggest that NO levels and bioavailability are markedly reduced in the atria during AF. Endocardial nitric oxide synthase (eNOS) expression and NO bioavailability are both reduced in the left atrium (but not the right atrium or aorta) after rapid atrial pacing in animal models, most likely as a result of a decrease in eNOS activity under conditions of turbulent blood flow [22]. The NO-cGMP pathway seems involved in the cardio-suppressive effects catestatin produces [20]. Catestatin inhibits the adrenergic-induced cardiostimulatory effect in both frogs and rats through noncompetitive antagonism and endothelin-1 (ET-1)-dependent mechanisms [23,24]. It was determined that catestatin acts as an endogenous NO-dependent blocker, which counteracts the inotropy increases induced by adrenergic and ET-1 stimulation [24]. The levels of cAMP, and thus the degree of PKA and Epac activation, are finely regulated by cyclic nucleotide phosphodiesterases (PDEs). It was reported that there is reduced total phosphodiesterases (PDE) and total PDE (IBMX-inhibited PDEs) activity in AF [25]. In contrast to vasostatin 1 (VS-1), the catestatin induced requires phosphodiesterases type 2 activation and the S-Nitrosylation of phospholamban and B-arrestin, which are essential for myocardial calcium modulation and B-adrenergic sensitivity, respectively [26]. It was recently determined that the cardio-suppressive peptides (VS-1) and catestatin, derived from CgA, act as endogenous NO-dependent B-blockers to counteract the adrenergic- and ET-1-mediated increases in inotropy [27]. 

Our results have shown that patients who take higher doses of beta blockers have higher levels of catestatin. Since patients receiving higher doses of beta blockers have more neurohumoral and SNS activation, and thus requiring more aggressive rate control, we thought their serum catestatin levels would be higher in comparison to patients receiving lower doses. The leakage of sarcoplasmic reticulum (SR) Ca^2+^ due to ryanodine receptor 2 (RyR2) PKA hyperphosphorylation may contribute to the development and/or sustenance of atrial fibrillation [28]. The spatial distribution of beta-adrenergic receptors associated with RyR2 is distinct from those associated with phospholamban (PLB) and sarcoplasmic endoplasmic reticulum calcium ATPase (SERCA2a). At higher concentrations, beta blockers were shown to affect not only RyR2 gating but also (PLB) phosphorylation (SERCA) activity and SR calcium content [29]. Catecholamine stimulation leads to phosphorylation of PLB and increased cardiac contractility, both of which require functional B1ARs at the SR [30]. Studies in a canine model of heart failure show that beta-adrenergic receptor blockers improve the structure and function of the ryanodine receptor (a cardiac calcium release channel) by reversing PKA hyperphosphorylation of RyR2, restoring the stoichiometry of the RyR2 macromolecular complex, and normalizing single-channel function [31]. Consistent with these findings, this new research shows that chronic treatment with B-AR blockers can normalize RyR2 complex composition and function in human patients with heart failure (HF) [32]. Catestatin’s antagonistic inotropic and lusitropic effects on the perfused Langendorff rat heart depend on its interaction with B2/B3-adrenergic receptors (B2/B3-AR), with a greater affinity for B2-AR [20]. Interestingly, the instability theory of Ca^2+^ handling has recently been challenged, raising the possibility that Ca^2+^ -induced calcium release may mediate AF in structurally remodeled atria, demonstrating how altered autonomic tone can cause AF [33,34,35]. In canine PVs, sympathetic activation induced an increase in myocardial cytoplasmatic (Ca^2+^), which was required for early afterdepolarizations in PVs and consequently triggered AF [36]. In vitro studies have shown that chronic catestatin treatment reduces AF vulnerability in rats with myocardial infarction (MI)-induced HF by improving Ca^2+^ handling through preserved SR Ca^2+^-ATPase protein expression but reducing the protein levels of phosphorylated-ryanodine receptor two and phosphorylated-Ca^2+^/calmodulin-dependent protein kinase II in the atria [37]. Exogenous catestatin enhanced autonomic function, decreased QT interval and action potential duration, and reduced experimentally induced ventricular arrhythmias in a rat model of myocardial infarction [38]. Elevated catestatin levels may arise from the body’s attempt to balance the consequences of increased SNS activity and excessive catecholamine release, although this may appear counterintuitive initially. Furthermore, it is conceivable that catestatin serum levels may be closely correlated with the catecholaminergic environment because catecholamines are co-stored and co-released from storage vesicles in adrenal chromaffin cells and adrenergic neurons with a group of acidic secretory proteins (such as ChgA) [39,40]. In this regard, catestatin levels may potentially represent catecholamine cycling and sympathetic activity in patients with AF. These findings provide support for the hypothesis that the effects of the beta-blocker drugs in higher dosages could be explained by their potential to reverse, in line with higher catestatin levels, the maladaptive response to the chronic hyperadrenergic state and thereby try to return the Ca^2+^ signaling to a normal physiological mode.

Although most patients with AF experience symptoms, the severity and the degree to which they affect patients’ functional status and quality of life (QoL) vary significantly from patient to patient [41,42]. Regardless of the type of AF, the EHRA score is regarded as one of the strongest indicators of QoL [43]. Palpitations, shortness of breath during exertion, and exhaustion are common AF symptoms [44]. Catestatin was positively associated with EHRA symptom score severity in our study. Anxiety has previously been linked as the strongest predictor of arrhythmia-related symptoms to AF, even though it is unclear whether it is the cause of AF-related symptoms or a result of AF [45]. A recent study concluded that anxiety positively correlated with plasma ChgA and catestatin levels [46]. Low-grade inflammation, assessed by high-sensitivity C-reactive protein (hs-CRP) levels, was found to predict a significant proportion of symptom variation in patients with AF [47]. Another recent study proposed the high-frequency contraction of atrial myocytes resulting in local inflammation as a possible additional stimulus in patients with AF. Low-grade inflammation could be a sign of longer, more active AF or the presence of other comorbidities that need to be treated to relieve AF symptoms [48]. Because of its ability to activate angiotensin-converting enzyme-2 (ACE2) and inhibit tumor necrosis factor alpha (TNF-alpha)-elicited expression of inflammatory cytokines and adhesion molecules, catestatin has been proposed as an anti-atherogenic and anti-inflammatory peptide that decreases leukocyte–endothelial interaction [49]. According to Chen et al., decreased catestatin leads to inflammation and thrombus development, while restoring catestatin prevents this by lowering endothelial inflammation [50]. Catestatin might reduce inflammation by reducing immune infiltration into inflamed tissues and shifting macrophage differentiation toward an anti-inflammatory phenotype. As a result, catestatin can reduce the severity of inflammatory responses and prevent tissue damage [51]. In accordance with our results, it was found in a recent CATSTAT-HF study that patients in a higher NYHA functional class had higher serum catestatin levels [52]. Our findings agree with a prior study showing an inverse relationship between serum catestatin levels and disease severity [53,54]. More recently, Martins et al. reported that long-term AF with a duration of up to 1 year and transition to persistent arrhythmia was accompanied by progressively increasing atrial dilation, mitral valve regurgitation, myocyte hypertrophy, and atrial fibrosis [55]. In conditions where cardiovascular homeostasis is disrupted, such as myocardial infarction, decompensated HF, and long-lasting AF, we hypothesize that the relatively high circulating catestatin levels are likely pathophysiologically overpowered by neurohumoral activity, minimizing the cardioprotective effects of catestatin. We believe this compensatory mechanism of catestatin may not be enough to stop the remodeling of the atrium in people with AF that lasts for a long time. Accordingly, it is plausible that elevated catestatin levels in highly symptomatic AF patients were an attempt at a compensatory cardioprotective response as part of the broader neurohormonal response to AF. 

CHA2DS2-VASc scores estimate yearly stroke risk and anticoagulant therapy indications and expert needs to be aware of new factors that can modify stroke [56,57,58]. Current guidelines recommend planning the anticoagulation treatment individually, according to the patient’s thromboembolic risk [1]. Several facts may be attributed to an apparent association between serum catestatin concentrations and CHA2DS2-VASc scores. For a start, accumulating data suggest that catestatin has a role in the pathogenesis of arterial hypertension. Primary hypertension (PH) patients have recently been reported to have greater serum catestatin concentrations than healthy controls [59]. Furthermore, previous research indicated that plasma catestatin levels in patients with moderate to severe HF were significantly increased [52,60]. In HF, interstitial fibrosis, myocyte hypertrophy, and oxidative stress are brought on by chronic and persistent stimulation by catecholamines, which reduces the responsiveness and function of cardiac-adrenergic receptors [61]. Zhu et al. found that as HF progressed from stage A to stage C, catestatin levels decreased [62]. Accordingly, our data indicated that serum catestatin concentrations inversely correlated with the time spent in AF. There is no clear explanation for this phenomenon at present. In animal research, CgA was hyperglycosylated in failing myocardium, and in clinical investigations, patients with little CgA-to-catestatin conversion had a worse result. They assumed myocardial glycosylation was greater in failing myocardium; it reflected posttranslational protein modification, and sugar groups could stop proteases from cleaving glycosylated proteins [63]. Finally, it has been established that serum catestatin positively correlates with age [64]. Therefore, higher serum catestatin levels may imply an increased neurohumoral burden in patients with a higher CHA2DS2-VASc score because all components of the CHA2DS2-VASc score are significant cardiovascular risk factors and related to increased catestatin levels.

We found that a higher serum concentration of catestatin was linked to a higher level of NT-proBNP. It has been shown that increasing levels of both angiotensin II (Ang II) and sympathetic tones cause a rise in NT-proBNP levels [65]. Patients with several comorbidities may have considerably high NT-proBNP levels that are somewhat prognostic despite the lack of considerable clinical indications of volume overload or left ventricular (LV) dysfunction [66]. The catestatin inhibition of catecholamine inhibits the Ang II-induced cell proliferation and the extracellular matrix (ECM) downregulation, regulating nitric oxide (NO) and the reactive oxygen species (ROS) pathways via inhibiting catecholamine release to protect the heart from fibrosis and atrial remodeling [67,68,69]. Januzzi et al. investigated the relationship between NT-proBNP levels, heart failure severity, and the diagnostic cut points for NT-proBNP in heart failure. Extreme heart failure and a poor prognosis were linked to values exceeding 1000 pg/mL [70]. The fact that our results are similar to those of studies of people with HF suggests that catestatin may be able to predict poor cardiac outcomes. At the same time, patients with elevated NTproBNP and catestatin levels were shown to have the highest mortality risk for any cause and cardiac causes. They found that serum catestatin levels provide additional information to the existing chronic HF prognostic factors [71]. Higher levels of NT-proBNP were associated with an increased risk of thromboembolic events and cardiovascular mortality in AF patients, supporting the function of NT-proBNP as a predictive marker for these outcomes [72]. As a marker for the development of atrial thrombi, higher NT-proBNP levels in AF may be linked to atrial dysfunction, explaining the observed correlation [73]. Plasma endothelin-1 (ET-1) levels are increased in permanent AF and HF when compared with HF patients in sinus rhythm, and atrial fibroblasts proliferate more upon stimulation with ET-1 when compared with ventricular fibroblasts [74,75]. Our results showed no statistically significant differences in catestatin levels between the three LVEF phenotypes. However, there is a trend for HFrEF patients to have higher catestatin levels. In the meantime, the sample size was too small to reach statistical significance. A recent study reported similar findings between HFrEF and HFpEF patients [60,76]. It makes sense that these two peptides, each associated with a different mechanism, would interact with one another in these patient populations given that a proposed mechanism of the relationship between catestatin and level of NT-proBNP is that AF causes changes in atrial volume, pressure, and wall stretch in accordance with neurohormonal activation. 

There are several limitations to this study. The study was designed as a single-center study and only included a Caucasian population. Hence, the applicability for other AF populations is limited. As the study was cross-sectional, no conclusions about causality can be drawn. On the other hand, as far as we know, this is the first study that looks at catestatin in people with AF and healthy controls. Understanding and treating AF more effectively may be possible by identifying the pathophysiological phenomena of atrial remodeling.

## 5. Conclusions

Based on the results of the present study, it is plausible that catestatin could be a good indirect indicator of an AF patient’s high sympathoexcitatory profile. Specifically, high serum catestatin concentrations could indicate advanced disease, and the presence of symptoms in patients with AF. Moreover, catestatin could give AF patients additional prognostic information about the risk of thromboembolic events and help with risk stratification. Finally, people with high levels of catestatin might be good candidates for starting or increasing beta-adrenergic blockers or other sympatholytic drugs. Nevertheless, all these findings are preliminary and must be addressed in larger prospective studies. 

## Figures and Tables

**Table 1:** Baseline characteristics of patients.

Parameter	Total (n = 145)	AF (n = 73)	Controls (n = 72)	*p*
Age, years	63 (57–70)	64 (59–72)	63 (55–69)	0.060 *
Male sex, n (%)	86 (59.3%)	47 (64.4%)	39 (54.2%)	0.212 ^†^
BMI, kg/m^2^	28.1 (25.9–31.0)	28.5 (26.0–31.2)	27.6 (25.7–30.4)	0.206 *
Hypertension, n (%)	82 (56.6%)	44 (60.3%)	38 (52.8%)	0.075 ^†^
Hemoglobin, g/L	149.1 ± 15.1	150.3 ± 14.9	146.7 ± 15.4	0.253 ^‡^
Total cholesterol, mmol/L	5.4 (4.4–6.3)	5.0 (4.1–6.1)	5.6 (4.6–6.3)	0.027 *
LDL, mmol/L	3.2 (2.3–4.0)	2.8 (2.1–4.0)	3.5 (2.7–4.1)	0.014 *
HDL, mmol/L	1.4 (1.2–1.7)	1.3 (1.1–1.7)	1.5 (1.3–1.6)	0.086 *
Triglycerides, mmol/L	1.3 (0.9–1.9)	1.4 (1.1–1.9)	1.1 (0.7–1.9)	0.026 *
CRP, mmol/L	1.7 (0.8–3.4)	2.0 (1.3–3.9)	1.3 (0.5–2.7)	0.005 *
NT-proBNP, ng/L	-	543 (208–894)	-	-
AF type				
Paroxysmal, n (%)	-	26 (35.6%)	-	-
Persistent, n (%)	-	34 (46.6%)	-	-
Long-persistent, n (%)	-	13 (17.8%)	-	-
AF duration, months	-	60 (24-120)	-	-
EHRA score				
1	-	2 (2.7%)	-	<0.001 ^†^
2	-	8 (11.0%)	-	
3	-	28 (38.4%)	-	
4	-	35 (47.9%)	-	
CHA2DS2-VASc score				
≤2	-	40 (54.8%)	40 (55.6%)	0.040 ^†^
>2	-	33 (45.2%)	32 (44.4%)	
HAS-BLED score				
0	-	37 (50.7%)	-	<0.001 ^†^
1	-	33 (45.2%)	-	
2	-	2 (2.7%)	-	
3	-	1 (1.4%)	-	
LA diameter, mm	-	45.1 ± 6.1	-	-
LVEF, %	-	60.5 (55.5-65.0)	-	-
Current antiarrhythmic therapy, n (%)	-	45 (61.6%)	-	-
Beta blockers, n (%)	-	44 (60.3%)	-	-
Statins, n (%)	-	25 (34.2%)	-	-

Data presented as mean ± SD, median (IQR) or n (%). * Mann–Whitney U test ^†^ Chi squared test ^‡^ Student’s *t*-test. Abbreviations: BMI: body mass index; CRP: C-reactive protein; AF: atrial fibrillation; NT-proBNP: N-terminal pro brain natriuretic peptide; EHRA: European heart rhythm association; LA: left atrium; LVEF: left ventricular ejection fraction.

## Data Availability

The data presented in this study are available on request from the corresponding author. The data are not publicly available because some of the data set will be used for further research.
